# Social support and prenatal mental health problems: a systematic review and meta-analysis

**DOI:** 10.1192/j.eurpsy.2022.300

**Published:** 2022-09-01

**Authors:** A. Tilahune, W. Peng, J. Adams, D. Sibbritt

**Affiliations:** University of Technology Sydney, Public Health, Sydney, Australia

**Keywords:** Depression, social support, Pregnancy, Anxiety

## Abstract

**Introduction:**

Pregnancy is a time of profound physical and emotional change as well as an increased risk of mental health problems. Providing social support is vital to reduce such risk.

**Objectives:**

This systematic review and meta-analysis aimed at examining the relationship between social support and depression, anxiety and self-harm during pregnancy.

**Methods:**

We searched observational studies from PubMed, Psych Info, MIDIRS, SCOPUS, and CINAHL databases. The Newcastle-Ottawa Scale tool was used for quality appraisal. The Q and the I² statistics were used to evaluate heterogeneity. A random-effects model was used to pool estimates. Publication bias was assessed using a funnel plot and Egger’s regression test and adjusted using trim and Fill analysis. All the analysis was conducted using STATA.

**Results:**

Sixty-seven studies with 64,449 pregnant women were part of the current review. Of the total 67 studies, 22 and 45 studies were included in the narrative analysis and meta-analysis, respectively. From the studies included in the narrative analysis, 20(91%) of them reported a significant association between social support and the risk of mental health problems (i.e. depression, anxiety, and self-harm). After adjusting for publication bias, the results of the random-effect model revealed low social support was significantly associated with antenatal depression (AOR: 1.18, 95% CI: 1.01, 1.41) and antenatal anxiety (AOR: 1.97, 95% CI: 1.34, 2.92).

**Conclusions:**

Low social support was significantly associated with depression, anxiety, and self-harm during pregnancy. Policy-makers and those working on maternity care should consider the development of targeted social support programs to help reduce mental health problems amongst pregnant women.

**Disclosure:**

No significant relationships.

